# Epidemiology of anaemia in children, adolescent girls, and women in Bhutan

**DOI:** 10.1111/mcn.12740

**Published:** 2018-11-29

**Authors:** Rebecca K. Campbell, Víctor M. Aguayo, Yunhee Kang, Laigden Dzed, Vandana Joshi, Jillian L. Waid, Suvadra Datta Gupta, Nancy Haselow, Keith P. West,

**Affiliations:** ^1^ Center for Human Nutrition, Department of International Health Johns Hopkins Bloomberg School of Public Health Baltimore Maryland; ^2^ Programme Division UNICEF Nutrition Programme New York New York; ^3^ Ministry of Health Government of Bhutan Thimphu Bhutan; ^4^ UNICEF Thimphu Bhutan; ^5^ Helen Keller International Dhaka Bangladesh; ^6^ Regional Office for Asia Helen Keller International Phnom Pehn Cambodia

**Keywords:** adolescent girls, anaemia, children, pregnant women, risk factors, South Asia

## Abstract

Anaemia inhibits health and development in Bhutan. We estimated anaemia prevalence and explored risk factors in children and women using data from Bhutan's National Nutrition Survey 2015. Prevalence was calculated using life‐stage‐specific cut‐offs adjusted for altitude and survey design. Risk factors were evaluated in modified Poisson regressions. Anaemia affected 42%, 29%, 36%, and 28% of children, adolescent girls, and non‐pregnant and pregnant women, respectively. Risk of anaemia was greater in children who were younger (RR 2.0, 95% CI [1.7, 2.3] and RR 1.9, 95% CI [1.6, 2.3], respectively, for 12–23 and 6–11 vs. 24–59 months), male (1.2, 1.1–1.4, ref.: female), and stunted (1.2, 1.0–1.3, ref.: height‐for‐age ≥ −2z). Older (15–19 years) versus younger (10–14 years) adolescents were at higher risk (1.5, 1.2–1.8), as were adolescents living at home versus at school (1.2, 0.9–1.6) and those working versus studying (1.3, 1.0–1.7). Among adult women, anaemia risk increased with age (1.2, 1.0–1.4 and 1.3, 1.1–1.5, for 30–39 and 40–49, respectively, vs. 20–29 years) and was higher for women without schooling (1.1, 1.0–1.3, vs. primary schooling), who were unmarried or separated (1.4, 1.2–1.7 and 1.3, 1.1–1.6, respectively, vs. married), without a child <5 years (1.1, 1.0–1.3), and lacking improved sanitation (1.1, 1.0–1.3). High coverage of antennal iron and folic acid supplementation may contribute to the lower prevalence of anaemia among pregnant women and women with young children. Expansion of iron supplementation programmes, fortification, and other strategies to improve dietary iron intake may reduce the prevalence of anaemia, but causes of anaemia other than iron deficiency (e.g., thalassemias) should also be investigated.

**Key messages**

- In Bhutan, the prevalence of anaemia among young children, adolescent girls, and women is high despite overall food security and moderately low prevalence of childhood undernutrition.
- A few sociodemographic factors were associated with increased risk of anaemia: in children, younger age and male sex; in women, older age, no schooling, being unmarried, and not having a child <5 years old.
- The lower prevalence of anaemia among pregnant women may reflect high coverage of the national prenatal iron‐folic acid supplementation programme.
- Causes of anaemia aside from iron deficiency, including thalassemia, require further investigation to inform expansion of anaemia control.

## 1 INTRODUCTION

Anaemia continues to burden large segments of the global population. An estimated 29% of non‐pregnant women and 43% of preschool‐age children worldwide have anaemia (Stevens et al., 2013), and iron‐deficiency anaemia is the single largest cause of years lived with disability in children and adolescents worldwide (Global Burden of Disease et al., 2017). Between 1990 and 2013, the global prevalence of anaemia declined by 21% (from 33.3% to 27.0%; Kassebaum, 2018), but progress has been uneven, with differences by region and greater improvements among men relative to women and among adults relative to children, widening previously observed disparities in anaemia prevalence among regions, sexes, and age groups (Kassebaum et al., 2014).

Anaemia is a condition of insufficient erythrocyte haemoglobin content, in which persistent inadequate oxygen delivery impairs the function of body tissues and organs, with wide‐ranging consequences. Anaemia is associated with weakness, fatigue, reduced productivity, and inhibited immune function (Beard, 2001; Fernandez‐Gaxiola & De‐Regil, 2011). In pregnant women, anaemia may cause low birthweight and preterm birth (Rasmussen, 2001) and increase the risk of maternal mortality due to haemorrhage and other delivery complications (Sanghvi, Harvey, & Wainwright, 2010). In children, anaemia is associated with impaired cognitive development and possibly with motor development as well (Balarajan, Ramakrishnan, Özaltin, Shankar, & Subramanian, 2011; Sachdev, Gera, & Nestel, 2005). Iron deficiency is the most common cause of anaemia (Kassebaum, 2018), but other causes include parasitic and other infections, chronic inflammatory conditions, deficiencies of micronutrients other than iron, and genetic causes (Bates, McKew, & Sarkinfada, 2007; Williams & Weatherall, 2012). The proportion of observed anaemia attributable to iron deficiency is estimated to be just over 60% worldwide but differs by geographic region and country and by demographic group (Kassebaum, 2018), as well as with the degree of economic development and prevalence of inflammation (Petry et al., 2016). Recently reported prevalence of anaemia in South and Southeast Asia is 58% in preschool‐age children and 47% in non‐pregnant women, despite substantial reductions in the past 15 years (Stevens et al., 2013). Prior to 2015, only two national surveys had described the burden of anaemia in Bhutan (Bailey, Lubach, & Coe, 2004; Food and Nutrition Section Department of Public Health Ministry of Health, 2003), and no study had investigated the subnational distribution of anaemia or its risk factors. Additionally, little data exist globally describing anaemia prevalence and risk factors among adolescent girls. In 2015, a National Nutrition Survey (NNS) in Bhutan reported high rates of anaemia among children, adolescent girls, and non‐pregnant and pregnant women of reproductive age, despite indicators of relatively good household diet quality and child nutritional status (Campbell et al., 2018; Kang et al., 2018). In the present study, we aimed to determine the risk factors for anaemia among each of the demographic groups included in Bhutan's 2015 NNS.

## 2 METHODS

### 2.1 Study design

Data for the present analysis came from the 2015 NNS in Bhutan. The survey utilized a multistage sampling strategy to generate representative nutritional status estimates nationally, for three major regions (West, Central, and East) and for the urban and rural areas of the country. The survey report describes the design of the survey in detail (Nutrition Program, 2015). Briefly, two districts (Dzongkhags) were randomly selected from each of the three regions, within which smaller administrative areas (rural Chiwogs and urban enumeration areas) were randomly selected in proportion to the urban/rural makeup of the region. Within each small administrative area included in the sample, 12 households were randomly selected. In each selected household, a questionnaire was administered to determine household socio‐economic characteristics and household composition. Additional questionnaires were administered to all pregnant women in the household and to the mother of the youngest child aged 0–23 months, and length/height and weight were measured for all children aged 0–59 months. Haemoglobin was measured with a Hemocue 301 (HemoCue, Brea, CA, USA) in eligible household members: children aged 6–59 months, adolescent girls aged 10–19 years, and pregnant and non‐pregnant women aged 20–49 years. Adolescent girls from selected households who were attending school away from home were located at school and their haemoglobin measured. Altitude at each selected Chiwog or enumeration area was recorded. For adolescent girls residing in schools, the school's altitude was recorded.

### 2.2 Data management

All participants with haemoglobin data were included in this analysis. Children, adolescent girls, and non‐pregnant and pregnant women were analysed separately. Children included all boys and girls aged 6–59 months. Adolescent girls were non‐pregnant girls aged 10–19 years. Non‐pregnant women were those aged 20–49 years, and pregnant women were all currently pregnant women 15–49 years. For anaemia prevalence estimates, non‐pregnant women aged 15–19 years were included in both the adolescent girls and the non‐pregnant adult women groups, for comparability with survey data from other countries for women of reproductive age. Women and adolescents with missing pregnancy status data were omitted from the analysis.

Participants with implausible haemoglobin values (<4 or ≥18 g/dl) were omitted from the analysis (*n* = 33 children and *n* = 20 adolescent girls and women; Sullivan, Mei, Grummer‐Strawn, & Parvanta, 2008). Haemoglobin values were adjusted for administrative area altitude with the Centers for Disease Control and Prevention (CDC) continuous adjustment equation (1989). Altitude‐adjusted haemoglobin and anaemia variables were used for all analyses. Cut‐offs for anaemia were based on World Health Organization (WHO) guidelines (WHO, 2011). For children, anaemia was defined as mild (Hb < 11 g/dl), moderate (Hb < 10 g/dl), and severe (Hb < 7 g/dl). For non‐pregnant adolescents and adult women, mild was Hb < 12 g/dl or Hb < 11.5 g/dl for girls 10 or 11 years old; moderate was Hb < 11 g/dl; and severe was Hb < 8 g/dl. For pregnant women, cut‐offs were mild (Hb < 11 g/dl), moderate (Hb < 10 g/dl), and severe (Hb < 7 g/dl).

Individual and household characteristics were measured by questionnaire. Improved sanitation was defined as the household having access to a private improved latrine, whereas improved water source was defined as having piped water into the house, the criterion set by the Government of Bhutan. A household wealth index was generated from household asset ownership and house structure variables according to a method previously developed by the Bhutan National Statistics Bureau. Food insecurity was assessed with a series of questions about the household's experience over the previous 6 months and was dichotomized for this analysis as any—one or more questions answered in the affirmative—or none. A food consumption score was calculated based on reported household food group consumption in the week before the survey and rated as acceptable, borderline, or poor (World Food Programme, 2008); borderline and poor were combined for this analysis. Household‐reported past‐week consumption of flesh foods (meat, fish, and eggs) was also examined separately, with reported intake categorized as none, 1 to 6 of the past 7 days, or daily. Anthropometry was measured in all children 0–59 months of age in selected households. Weight was measured to the nearest 0.1 kg using a portable Seca 881 scale (Chino, CA, USA). Length or height was measured to the nearest 0.1 cm using a wooden measuring board (Nutrition Program, 2015).

Adolescent girls' and women's ages were self‐reported in years, whereas children's ages were calculated from maternal‐reported date of birth. Adolescents' and adults' level of completed education was collected in categories: none, early learning, primary completed, high school completed, some university, diploma/certificate completed, monastic schooling, and non‐formal education. For adult women, the categories were combined into a three‐level scale: none or early learning only; primary, monastic, or informal schooling; high school, university, or diploma. For adolescents, three categories of education were considered: none, early learning, monastic, or informal schooling; primary school; and high school or greater. Women and adolescents reported their occupation in an open‐ended question. For adolescents, the responses were grouped into student; farmer or other employment; and no employment. For adult women, occupation responses were grouped into student, farmer, other employment, and housewife/other. Women's marital status was recorded as never married, living together, married, divorced, separated, or widow. For this analysis, living together and married were combined into one group labelled “married,” and divorced, separated, and widowed were combined into a “separated” group.

### 2.3 Statistical analysis

National, regional, and urban/rural prevalence of anaemia was estimated for each demographic group using the “svy:” prefix to account for survey weights and clustering of observations from the design of the survey. For each group, prevalence of mild, moderate, and severe anaemia were estimated separately, as was prevalence of any anaemia with mild, moderate, and severe combined.

Models of individual and household factors predictive of anaemia were developed for the demographic groups separately using modified Poisson regression using the generalized linear model command with robust standard errors (Zou, 2004). For each demographic group, unadjusted regression models with dichotomous anaemia status as the outcome were generated for each potential risk factor. All variables significant at the *p* ≤ 0.10 level were then included in a multivariable model along with known anaemia risk factors: age, sex (child model only), region, urban/rural location, and household wealth index. Multivariable regression models were developed in the same way for each demographic group with independent variable continuous altitude‐adjusted haemoglobin as a sensitivity analysis for the influence of the anaemia cut‐offs on observed associations. A block‐stepwise modelling approach was also applied for dichotomous and continuous outcomes, as a sensitivity analysis, to check that significant predictors in a fully adjusted model were not overlooked in a data‐driven modelling approach (Engle‐Stone et al., 2017). For this sensitivity analysis, blocks of risk factors were selected by referencing the conceptual framework proposed in Namaste et al. (2017). Survey‐adjusted models were also developed using the “svy:” command with Poisson models in a further sensitivity analysis of the effect of the survey sampling strategy on the anaemia risk factors identified. All analyses were conducted in Stata 14.1 (StataCorp, College Station, TX, USA).

## 3 RESULTS

Haemoglobin data were available for 1,083 children aged 6–59 months, 1,216 non‐pregnant adolescent girls aged 10–19 years, 2,649 non‐pregnant women aged 20–49 years, and 118 pregnant adolescents and women aged 15–49 years living in 2,478 households. Most children, adolescents, and women included in the sample lived in rural households (75%). Most households reported improved sanitation facilities (70%) and piped drinking water (86%). Household food insecurity and poor or borderline food consumption scores were reported infrequently (2% and 8%, respectively), and many households reported consuming flesh foods daily (68%). About 40% of households were located in administrative areas at altitudes between 1,000 and 2,000 m, and an additional 26% were located at altitudes ≥2,000 m.

### 3.1 Anaemia prevalence

In Table 1, estimates of anaemia prevalence adjusted for survey design are presented. In all groups, anaemia prevalence was high. Among children aged 6–59 months, 42% were anaemic: 25% had mild anaemia (10 g/dl ≤ Hb < 11 g/dl), 17% had moderate anaemia (7 g/dl ≤ Hb < 10 g/dl), and fewer than 1% had severe anaemia (Hb < 7 g/dl). The prevalence of anaemia among adolescent girls was 29%: 15% had mild anaemia (11 g/dl ≤ Hb < 12 g/dl; 11 g/dl ≤ Hb < 11.5 g/dl for girls 10–11 years), 13% had moderate anaemia (8 g/dl ≤ Hb < 11 g/dl), and 1% had severe anaemia (Hb < 8 g/dl). In non‐pregnant women 15–49 years, the prevalence of anaemia was 36%, split evenly between mild anaemia (18%) and moderate anaemia (17%), with an additional 1% severely anaemic. The prevalence of anaemia among pregnant women was 28%: 18% with mild anaemia and 10% with moderate anaemia.

**Table 1:** National and regional prevalence of anaemia in children, adolescents, and non‐pregnant and pregnant women in Bhutan

Demographic group	*N*	Anaemia, percent (*SE*)^a^			
		Any	Mild	Moderate	Severe
Children 6–59 months^b^	1,083	42.3 (3.3)	25.0 (1.4)	16.9 (2.5)	0.4 (0.3)
Adolescent girls 10–19 years^c^	1,216	29.3 (3.6)	15.1 (1.7)	12.9 (1.9)	1.3 (0.4)
Non‐pregnant women 15–49 years^d^	3,233	36.3 (2.1)	18.0 (0.8)	16.9 (1.3)	1.4 (0.3)
Pregnant women 15–49 years^e^	118	28.2 (3.4)	17.9 (3.5)	10.3 (3.2)	0 (0)

a Estimates adjusted for survey design using “svy:” commands.

b For children, mild anaemia (Hb < 11 g/dl), moderate anaemia (Hb < 10 g/dl), and severe anaemia (Hb < 7 g/dl).

c For non‐pregnant adolescent girls, mild anaemia (Hb < 12 g/dl; Hb < 11.5 g/dl for girls 10–11 years), moderate anaemia (Hb < 11 g/dl), and severe anaemia (Hb < 8 g/dl).

d For non‐pregnant women, mild anaemia (Hb < 12 g/dl), moderate anaemia (Hb < 11 g/dl) and severe anaemia (Hb < 8 g/dl).

e For pregnant women, mild anaemia (Hb < 11 g/dl), moderate anaemia (Hb < 10 g/dl), and severe anaemia (Hb < 7 g/dl).

### 3.2 Risk factors for anaemia

Table 2 presents multivariable models for anaemia risk factors in young children, in which child age, sex, and stunting status were associated with risk of anaemia. Children 6–11 and 12–23 months old (RR: 1.9, 95% CI: 1.6–2.3 and 2.0, 1.7–2.3, respectively, ref: 24–59 months) and boys (1.2, 1.1–1.4, ref: girls) were at greater risk of anaemia. Stunted children (1.2, 1.0–1.3, ref: height‐for‐age ≥ −2z) were also at a higher risk of anaemia. Odds ratios for anaemia in children with wasting and overweight were both positive but with 95% confidence intervals that included unity (1.1, 0.9–1.5 and 1.3, 0.9–1.8, respectively). Risk of anaemia differed somewhat by region of the country, with children in the West at somewhat higher risk of anaemia compared with those in the East (1.1, 1.0–1.3). Most household socio‐economic and diet quality markers were not associated with anaemia, although living in a household without improved sanitation or improved water was each associated with a trend towards increased risk of anaemia (1.1, 0.9–1.2 and 1.1, 0.9–1.3 for sanitation and water, respectively). Associations between infant and young child feeding indicators and anaemia status in children <2 years were evaluated, but no associations were observed (results not shown). In multivariable models with haemoglobin concentration as a continuous outcome, significant risk factors were similar to those in dichotomous anaemia models. In addition, having a mother with moderate anaemia was associated with 0.4 g/dl (−0.7 to −0.2) lower Hb concentration (not shown).

**Table 2:** Personal and household characteristics predictive of children's risk of anaemia (*n* = 1,083)

Characteristic^a^ [Link]	*N*	Hb (g/dl), mean (*SD*)	RR (95% CI)^b^ [Link]
Region			
East	481	11.5 (1.4)	1.0
West	311	11.7 (1.2)	1.1 (1.0, 1.3)
Central	291	11.6 (1.2)	1.0 (0.8, 1.2)
Area			
Urban	232	11.6 (1.2)	1.0
Rural	851	11.6 (1.3)	0.9 (0.8, 1.2)
Wealth quintiles			
Highest	92	11.7 (1.2)	1.0
High	145	11.5 (1.2)	1.0 (0.7, 1.4)
Medium	196	11.5 (1.4)	1.1 (0.8, 1.5)
Low	312	11.6 (1.3)	1.2 (0.9, 1.7)
Lowest	338	11.6 (1.4)	1.1 (0.8, 1.6)
Improved sanitation			
Yes	721	11.6 (1.3)	1.0
No	362	11.4 (1.3)	1.1 (0.9, 1.2)
Improved water			
Yes	924	11.6 (1.3)	1.0
No	159	11.4 (1.3)	1.1 (0.9, 1.3)
Child age, months			
24–59	709	11.8 (1.2)	1.0
12–23	258	11.1 (1.3)	2.0 (1.7, 2.3)
6–11	116	11.1 (1.3)	1.9 (1.6, 2.3)
Child sex			
Female	575	11.7 (1.3)	1.0
Male	508	11.4 (1.4)	1.2 (1.1, 1.4)
Stunted^c^ [Link]			
No	799	11.6 (1.3)	1.0
Yes	256	11.5 (1.3)	1.2 (1.0, 1.3)
Wasted^c^ [Link]			
No	1,017	11.6 (1.3)	1.0
Yes	41	11.3 (1.3)	1.1 (0.9, 1.5)
Overweight^c^ [Link]			
No	1,059	11.6 (1.3)	1.0
Yes	24	10.8 (1.3)	1.3 (0.9, 1.8)

^a^Only personal/household characteristics that were included in the multivariable model are listed in the table. Variables that were investigated but were not significant were as follows: household food insecurity, food consumption score, and meat consumption; maternal education, age, and anaemia; and child underweight.

^b^Risk ratios and confidence intervals generated with modified Poisson regression models with robust standard errors.

^c^Dichotomous anthropometry indicator definitions: stunted: HAZ or LAZ < −2; wasted: WLZ or WHZ < −2; overweight: WLZ or WHZ > 2.

Table 3 presents risk factors for anaemia among adolescent girls, in whom older age (15–19 vs. 10–14 years) was associated with increased risk of anaemia (1.5, 1.2–1.8), as was working as a farmer or in another occupation (1.3, 1.0–1.7) versus being a student. Living in the West or Central region was associated with increased risk of anaemia (1.3, 1.1–1.7 and 1.3, 1.0–1.6 for West and Central, respectively, relative to East), and living at home was also associated with a greater anaemia risk relative to residing at school, although the confidence interval included 1 (1.2, 0.9–1.6). Household socio‐economic status (SES) and diet quality indicators were not predictive of adolescent girls' risk of anaemia. In models for continuous Hb, the only additional risk factor that emerged was the availability of improved water source in the household, which was surprisingly associated with 0.3 (−0.5 to −0.1) g/dl lower Hb (not shown).

**Table 3:** Personal and household characteristics predictive of adolescent girls' risk of anaemia (*n* = 1,216)

Characteristic^a^ [Link]	*n*	Hb (g/dl), mean (*SD*)	RR (95% CI)^b^ [Link]
Region			
East	521	13.0 (1.4)	1.0
West	350	13.0 (1.6)	1.3 (1.1, 1.7)
Central	345	12.8 (1.5)	1.3 (1.0, 1.6)
Area			
Urban	305	12.6 (1.5)	1.0
Rural	911	13.0 (1.4)	1.0 (0.8, 1.3)
Wealth quintiles			
Highest	145	12.8 (1.4)	1.0
High	150	12.6 (1.5)	1.2 (0.8, 1.6)
Medium	207	12.9 (1.4)	0.9 (0.6, 1.3)
Low	370	13.0 (1.6)	1.0 (0.7, 1.4)
Lowest	344	13.0 (1.3)	1.0 (0.7, 1.5)
Age, years			
10–14	631	13.0 (1.3)	1.0
15–19	585	12.8 (1.6)	1.5 (1.2, 1.8)
Occupation			
Student	1,045	13.0 (1.4)	1.0
Farmer/other	109	12.7 (1.6)	1.3 (1.0, 1.7)
None	62	12.8 (1.6)	1.3 (0.9, 1.9)
Residence			
School	225	13.3 (1.4)	1.0
Home	991	12.8 (1.5)	1.2 (0.9, 1.6)

^a^Only personal/household characteristics that were included in the multivariable model are listed in the table. Variables that were investigated but were not significant were as follows: education and marital status; and household improved sanitation, improved water, food insecurity, food consumption score, and meat consumption.

^b^Risk ratios and confidence intervals generated with modified Poisson regression models with robust standard errors.

Table 4 presents risk factors for anaemia among non‐pregnant adult women. The risk of anaemia was associated with region, household sanitation, women's age, level of education, marital status, and maternal status. Living in the West or Central region was associated with greater anaemia risk (1.5, 1.3–1.7 and 1.4, 1.2–1.6, respectively, relative to East), as was living in a house without an improved sanitation facility (1.1, 1.0–1.3). Risk of anaemia increased with age (1.2, 1.0–1.4 and 1.3, 1.1–1.5 in women ages 30–39 and 40–49 years, respectively, relative to women 20–29 years). Having no schooling was associated with increased risk of anaemia (1.1, 1.0–1.3) relative to those who attended primary school, but the risk of anaemia among women who completed high school was not statistically significantly different from those who attended primary school. Unmarried and separated/divorced/widowed women relative to married (1.4, 1.2–1.7 and 1.3, 1.1–1.6, respectively) and those without a child under age 5 years (1.1, 1.0–1.3) were at greater risk of anaemia. Associations between risk factors and continuous Hb were similar (not shown).

**Table 4:** Personal and household characteristics predictive of non‐pregnant women's risk of anaemia (*n* = 2,649)

Characteristic^a^ [Link]	*n*	Hb (g/dl), mean (*SD*)	RR (95% CI)^b^ a
Region			
East	1,094	13.1 (1.7)	1.0
West	798	12.8 (1.7)	1.5 (1.3, 1.7)
Central	757	12.7 (1.7)	1.4 (1.2, 1.6)
Area			
Urban	648	12.7 (1.6)	1.0
Rural	2,001	12.9 (1.7)	1.0 (0.8, 1.1)
Wealth quintiles			
Highest	313	12.8 (1.6)	1.0
High	368	12.8 (1.6)	0.9 (0.7, 1.1)
Medium	506	12.8 (1.8)	1.0 (0.8, 1.2)
Low	791	13.0 (1.7)	1.0 (0.8, 1.3)
Lowest	671	12.9 (1.7)	1.0 (0.8, 1.3)
Improved sanitation			
Yes	1,910	12.9 (1.7)	1.0
No	739	12.7 (1.8)	1.1 (1.0, 1.3)
Age, years			
20–29	1,070	13.0 (1.6)	1.0
30–39	890	12.9 (1.7)	1.2 (1.0, 1.4)
40–49	689	12.7 (1.8)	1.3 (1.1, 1.5)
Education			
High school+	701	12.8 (1.6)	1.1 (0.9, 1.3)
Primary^c^ b	729	13.1 (1.6)	1.0
None	1,214	12.8 (1.8)	1.1 (1.0, 1.3)
Occupation			
Farmer	1,300	13.0 (1.8)	1.0
Student	146	12.7 (1.8)	0.9 (0.7, 1.2)
Other job	392	12.6 (1.7)	1.1 (0.9, 1.3)
Housewife/other	811	12.9 (1.6)	1.0 (0.9, 1.1)
Marital status			
Married	2,040	13.0 (1.7)	1.0
Unmarried	399	12.5 (1.9)	1.4 (1.2, 1.7)
Separated^d^ c	210	12.7 (1.8)	1.3 (1.1, 1.6)
Child <5 years^e^ d			
Yes	949	13.1 (1.5)	1.0
No	1,673	12.7 (1.8)	1.1 (1.0, 1.3)

^a^Only personal/household characteristics that were included in the multivariable model are listed in the table. Variables that were investigated but were not significant: household improved water, food insecurity, food consumption score, and meat consumption.

a ^b^Risk ratios and confidence intervals generated with modified Poisson regression models with robust standard errors.

b ^c^Primary category also includes informal and monastic schooling.

c ^d^Separated category includes divorced, separated, and widowed.

d ^e^Woman is the mother of a child age 5 years or younger.

Models for anaemia risk factors in pregnant women, shown in Table 5, were limited by the small sample size of pregnant women (*n* = 118). In multivariable models, only older age (3.6, 1.7–7.6 for those 0–49 vs. 20–29 years) and trimester of pregnancy (2.8, 1.2–6.2 and 2.7, 1.3–5.6 for second and third trimester, respectively, relative to first trimester) persisted as significant predictors of anaemia. Risk ratios for anaemia in lower wealth quintiles were also positive, though confidence intervals mostly included 1. No risk factors reached statistical significance in multivariable linear regressions for continuous Hb (not shown).

**Table 5:** Characteristics predictive of pregnant women's odds of anaemia (*n* = 118)

Characteristic^a^ [Link]	*n*	Hb (g/dl), mean (*SD*)	RR (95% CI)^b^
Region			
East	47	12.2 (1.4)	1.0
West	40	11.7 (1.2)	0.9 (0.5, 1.7)
Central	31	11.5 (1.4)	1.0 (0.5, 1.8)
Area			
Urban	32	11.9 (1.7)	1.0
Rural	86	11.7 (1.3)	0.6 (0.3, 1.5)
Wealth quintiles			
Highest	15	12.6 (1.6)	1.0
High	23	12.0 (1.0)	0.7 (0.2, 2.9)
Medium	22	11.2 (1.4)	3.3 (1.1, 9.6)
Low	26	11.3 (1.1)	2.7 (0.9, 8.0)
Lowest	32	11.8 (1.4)	1.6 (0.5, 5.2)
Age, years			
15–19	9	12.0 (0.6)	0.4 (0.1, 2.5)
20–29	67	11.8 (1.1)	1.0
30–39	34	12.6 (1.8)	1.0 (0.5, 2.0)
40–49	8	11.9 (1.5)	3.6 (1.7, 7.6)
Trimester			
1	42	12.6 (1.6)	1.0
2	44	11.6 (1.2)	2.8 (1.2, 6.2)
3	32	11.1 (1.1)	2.7 (1.3, 5.6)

^a^Only personal/household characteristics that were included in the multivariable model are listed in the table. Variables that were investigated but were not significant in unadjusted models were as follows: gravidity, receipt of any ANC, initiation of ANC in the first trimester of pregnancy and past 7‐day iron and folic acid supplement consumption.

^b^Risk ratios and confidence intervals generated with modified Poisson regression models with robust standard errors.

In models adjusted for survey design, magnitudes of risk associations generally persisted, but fewer confidence intervals excluded zero (Tables S3–S6).

## 4 DISCUSSION

This national survey in 2015 revealed the prevalence of anaemia in Bhutan to be 42%, 29%, 36%, and 28% among preschool‐age children, adolescent girls, non‐pregnant women, and pregnant women, respectively. In children and pregnant women, most anaemia was mild, whereas among adolescent girls and non‐pregnant women, about half of all anaemia cases were moderate in severity, although few cases of severe anaemia were observed. In 1985 and 2002, two national anaemia surveys among children under 5 years of age reported prevalence rates of 58% and 81%, respectively (Bailey et al., 2004). Although the former compares with anaemia rates in the region in 1990 (Kassebaum et al., 2014), reasons for the arguably implausible estimate from 2002 remain unclear. Our current finding of a decline to 42% in 2015, however, is consistent with a gradual trend of nutrition and health conditions that are improving in the country (Atwood, Napgal, Mbuya, & Laviolette, 2014; Tobgay, Dorji, Pelzom, & Gibbons, 2011). Trend data for adult women are less comparable, as the 1986 survey in Bhutan examined anaemia only in pregnant women whereas the 2002 survey reported anaemia only in non‐pregnant women. Neither survey included adolescents. Nonetheless, the observed high prevalence of anaemia in children and women in 2015 reveals a continued need to better understand its causes and risk factors to improve prevention.

Anaemia prevalence rates reported here for preschoolers and adult women in Bhutan were intermediate within those reported from other countries in South and Southeast Asia over the past two decades (Table S1). For adolescent girls in the region, only Afghanistan has comparable national data, where 30% were reported to have anaemia (Ministry of Public Health of the Islamic Republic of Afghanistan and UNICEF, 2013).

Risk of anaemia varied with age—it was greater in younger than older preschoolers and increased with age in adolescent girls and in adult women—and was higher in the Central and Western than Eastern regions, associations that may help guide causal investigations and prevention. Dietary patterns were not associated with haemoglobin concentration in preschool children, a surprising finding given that dietary diversity remains inadequate among young Bhutanese children (Campbell et al., 2018). Recent analyses of Demographic and Health Survey data in Bangladesh also failed to find associations between anaemia and the WHO‐defined indicator of a minimum acceptable diet (frequency and diversity of complementary foods; WHO, 2010) or reported intake of iron‐rich foods among children aged 6–23 months (Rawat et al., 2014). A lack of association could reflect non‐specificity of dietary quality indicators obtained in a cross‐sectional design in revealing usual dietary patterns that increase risk of iron deficiency or could point to a higher burden of causes of anaemia other than iron deficiency.

Household socio‐economic status was unrelated to risk of anaemia in preschool‐age children. These findings contrast with those of studies in low‐income settings of Bangladesh and Nepal, where SES was associated with risk of early childhood anaemia (Balarajan, Fawzi, & Subramanian, 2013; Khan, Awan, & Misu, 2016; Rohner et al., 2013), but agree with findings of population studies in Cambodia and Vietnam where links between anaemia and SES were not observed (Charles et al., 2015; Nguyen et al., 2006), possibly reflecting a lack of association under socio‐economic conditions with less extreme poverty (National Statistics Bureau, 2014). The suggestion that piped water may be associated with poorer Hb status, though not observed in models for dichotomous anaemia, is consistent with findings from Bangladesh showing better iron status in women in poorer, more rural areas who consume tube well water drawn from aquifers laden with iron relative to women in more urban areas who drink piped water (Merrill et al., 2011; Rahman et al., 2016). To our knowledge, no studies of groundwater iron in Bhutan have been conducted, but it could be an avenue for further investigation.

We did, however, observe the risk of anaemia to be higher among stunted preschool children; adolescent girls living at home rather than at school and those with employment rather than studying; and uneducated, unmarried or separated women, and mothers not having a preschool‐aged child. These risk factors possibly reflect, respectively, chronic undernutrition underlying anaemia risk in children, adolescent girls being protected somewhat by school‐based iron supplementation programmes that have been in place since 2004 (Ministry of Health and UNICEF, 2014), and perhaps durable effects of antenatal iron supplementation and other health system contact among women who had been pregnant recently. These observed relationships offer evidence of groups within the population who are at greater risk of anaemia, but modest risk ratios and the possibility of residual confounding should be considered.

The possibility that routine health care including iron‐folic acid (IFA) supplementation during pregnancy reduced anaemia risk in pregnant women is supported by the observation of nearly 20% lower anaemia prevalence among pregnant than non‐pregnant women (despite higher iron demands during pregnancy). This difference, combined with previously reported high national attendance at antenatal care (98%) and adherence to daily prenatal IFA supplementation (87%; Nutrition Program, 2015), suggests that regular IFA supplementation through the country's antenatal care system is effectively protecting many women from anaemia during pregnancy and may be building iron stores that confer extended postpartum protection against iron deficiency anaemia. However, the fact that gestational anaemia is not fully prevented with extensive IFA coverage may suggest that either the supplemental iron being delivered is still inadequate, other hematinic nutrient deficiencies are not being prevented (Fishman, Christian, & West, 2000) or causes other than iron deficiency contribute in many anaemia cases (e.g., thalassemia), as has been reported from Bangladesh (Merrill et al., 2012). Such causes are known to vary markedly by population (Balarajan et al., 2011), and the burdens of parasitic infections, other micronutrient deficiencies, and genetic haemoglobinopathies are not well described in Bhutan. Evidence from elsewhere in South and Southeast Asia, however, suggests that in certain subpopulations within the region, rates of hemoglobinopathies are very high (Fucharoen & Winichagoon, 2011; Hossain et al., 2017; Wah, Yi, Khin, Plabplueng, & Nuchnoi, 2017), appearing to warrant their assessment and public health management. Better understanding the contributions of thalassemia and other causes of anaemia in addition to iron deficiency could better guide efforts to reduce anaemia in Bhutan.

Assuming that there remains anaemia responsive to iron in Bhutan, several strategies exist to expand national delivery of iron across life stages. These include improving intakes of animal‐source and other iron‐rich foods, introduction of point‐of‐use fortification (Suchdev, Pena‐Rosas, & De‐Regil, 2015), fortification of processed foods or condiments (Das, Salam, Kumar, & Bhutta, 2013; Gera, Sachdev, & Boy, 2012) that penetrate rural markets and/or are targeted to children and women, or bio‐fortification of staple crops, which has been demonstrated effective in adults with rice, pearl millet, and beans in the Philippines, India, and Rwanda, respectively (Finkelstein, Haas, & Mehta, 2017).

The present study benefits from having analysed data from a nationally representative anaemia survey that allowed anaemia status to be linked to household and individual characteristics. Additionally, anaemia assessment in a large sample of young children, adolescent girls, and pregnant and non‐pregnant women of reproductive age is an unusual feature of an NNS that enabled prevalence, trends, and risk factors for anaemia to be examined across these multiple life stages. The lack of status data on iron, other micronutrients, and inflammation limited our ability to differentiate causes of anaemia. Additionally, the small sample of pregnant women limited the risk factor analysis in that group.

Bhutan's 2015 NNS reveals a setting of surprisingly high anaemia prevalence given the relatively good nutritional status in children and women. New and enhanced programmes are needed to prevent anaemia in preschool‐age children, adolescent girls, and women of reproductive age to protect the growth, development, and wellbeing of these vulnerable groups. Identification of few sociodemographic or nutritional/dietary risk factors for anaemia coupled with lack of data on iron deficiency, inflammation, and other aetiologies of anaemia warrants further investigation into causes of anaemia in Bhutan. Such data combined with the present findings may be expected to better inform the design and implementation of anaemia control strategies in children, adolescent girls, and women of reproductive age.

## CONFLICTS OF INTEREST

The authors declare that they have no conflicts of interest.

## CONTRIBUTIONS

RKC, VMA, YK, and KPW designed the study; VMA, LD, and VJ led the design and implementation of the NNS 2015; VMA, LD, VJ, JLW, SDG, and NH prepared and analysed the data for the survey report; RKC and YK conducted data analysis for this study; RKC wrote the manuscript. All authors read and approved the manuscript; KPW had primary responsibility for the final content.

## Supporting information


**Table S1**. Prevalence of anaemia by country and demographic group in the most recent available surveys for the countries of South and Southeast Asia
**Table S2**. Regional prevalence of anaemia in children, adolescents, non‐pregnant and pregnant women in Bhutan
**Table S3**. Personal and household characteristics predictive of children's risk of anaemia (Hb < 11 g/dL) in survey‐adjusted models (*n* = 1083)
**Table S4**. Personal and household characteristics predictive of adolescent girls' risk of anaemia (Hb < 12 g/dL, Hb < 11.5 g/dL for girls 10–11 y) in survey‐adjusted models (*n* = 1216)
**Table S5**. Personal and household characteristics predictive of non‐pregnant women's risk of anaemia (Hb < 12 g/dL) (*n* = 2649)
**Table S6**. Characteristics predictive of pregnant women's odds of anaemia (*n* = 118)
