# Supplementary material for: Epidemiology of anaemia in children, adolescent girls, and women in Bhutan
Source: Matern Child Nutr. 2018 Nov 29;14(Suppl 4):e12740. doi: 10.1111/mcn.12740 (PMC6948218; doi:10.1111/mcn.12740)
Supplement: Supplementary file 1 — Table S1. Prevalence of anaemia by country and demographic group in the most recent available surveys for the countries of South and Southeast Asia Table S2. Regional prevalence of anaemia in children, adolescents, non‐pregnant and pregnant women in Bhutan Table S3. Personal and household characteristics predictive of children's risk of anaemia (Hb < 11 g/dL) in survey‐adjusted models (n = 1083) Table S4. Personal and household characteristics predictive of adolescent girls' risk of anaemia (Hb < 12 g/dL, Hb < 11.5 g/dL for girls 10–11 y) in survey‐adjusted models (n = 1216) Table S5. Personal and household characteristics predictive of non‐pregnant women's risk of anaemia (Hb < 12 g/dL) (n = 2649) Table S6. Characteristics predictive of pregnant women's odds of anaemia (n = 118) [file MCN-14-e12740-s001.docx]

SUPPLEMENTARY APPENDIX

Supplemental Table 1. Prevalence of anemia by country and demographic group in the most recent available surveys for the countries of South and Southeast Asia

| **Country** | **Source** | **Survey Year** | **Children** | | **Women** | | | |
| --- | --- | --- | --- | --- | --- | --- | --- | --- |
|  |  |  | **Age (mo)** | **Prevalence (%)** | **Age (y)** | **Prevalence (%)** | | |
|  |  |  |  |  |  | **Overall** | **Not pregnant** | **Pregnant** |
| Bhutan | (Nutrition Program, 2015) | 2015 | 6-59 | 42.0 | 15-49 |  | 36.0 | 26.0 |
| Afghanistan | (Ministry of Public Health of the Islamic Republic of Afghanistan and UNICEF, 2013) | 2013 | 6-59 | 44.9 | 15-49 | 40.4 |  |  |
| Bangladesh | (National Institute of Population Research and Training (NIPORT) et al., 2013) | 2011 | 6-59 | 51.3 | 15-49 | 42.4 | 40.0 | 49.6 |
| Cambodia | (National Institute of Statistics/Cambodia et al., 2015) | 2014 | 6-59 | 55.5 | 15-49 | 45.4 | 43.8 | 53.2 |
| India | (International Institute for Population Sciences - IIPS/India and Macro International, 2007) | 2005-6 | 6-59 | 69.5 | 15-49 | 55.3 | 53.2 | 58.7 |
| Laos | (Ministry of Health [Lao People’s Democratic Republic], 2001) | 2000 | 0-72 | 48.2 |  |  |  |  |
| Myanmar | (Ministry of Health and Sports (MoHS) and ICF, 2017) | 2015-16 |  | 57.8 |  | 46.5 | 45.8 | 56.9 |
| Nepal | (Ministry of Health and Population - MOHP/Nepal et al., 2012) | 2011 | 6-59 | 46.2 | 15-49 | 35.0 | 33.0 | 47.6 |
| Pakistan | (Aga Khan University et al., 2011) | 2011 | 6-59 | 62.1 | 16-49 |  | 50.5 | 51.2 |
| Sri Lanka | (Department of Census and Statistics (DCS) and Ministry of Healthcare and Nutrition (MOH), 2009) | 2006-7 | 6-59 | 32.6 | 15-50 |  | 39.1 | 34.0 |
| Thailand | (Ministry of Public Health, 1998) | 1995 | 0-72 | 25.2 | 15-45 | 17.6 | 16.8 | 22.3 |
| Viet Nam | (Khoi HH et al., 2001) | 2000 | 0-59 | 34.1 | 15-50 |  | 24.3 | 32.2 |

Supplemental Table 2. Regional prevalence of anemia in children, adolescents, non-pregnant and pregnant women in Bhutan

|  | **Anemia, Proportion (SE)^a^** | | | |
| --- | --- | --- | --- | --- |
|  | **Any** | **Mild** | **Moderate** | **Severe** |
|  | **Children, 6-59 mo (n=1083)^b^** | | | |
| National | 42.3 (3.3) | 25.0 (1.4) | 16.9 (2.5) | 0.4 (0.3) |
| Region |  |  |  |  |
| West | 47.0 (6.0) | 27.3 (2.5) | 19.6 (4.1) | 0.1 (0.1) |
| Central | 37.4 (8.0) | 22.3 (1.9) | 15.0 (6.9) | 0.1 (0.1) |
| East | 40.4 (2.5) | 24.2 (2.2) | 15.2 (1.6) | 1.0 (0.6) |
| Area |  |  |  |  |
| Urban | 41.8 (6.4) | 26.5 (2.6) | 14.5 (4.8) | 0.7 (0.6) |
| Rural | 42.8 (4.4) | 23.5 (1.6) | 19.1 (3.4) | 0.1 (0.1) |
|  |  | | | |
|  | **Adolescent girls, 10-19 y (n=1216)^c^** | | | |
| National | 29.3 (3.6) | 15.1 (1.7) | 12.9 (1.9) | 1.3 (0.4) |
| Region |  |  |  |  |
| West | 33.6 (7.7) | 15.7 (3.8) | 14.8 (3.6) | 3.0 (0.9) |
| Central | 30.3 (4.1) | 15.7 (1.5) | 14.7 (3.2) | 0.0 (0.0) |
| East | 24.0 (2.0) | 14.0 (1.6) | 9.6 (1.2) | 0.4 (0.2) |
| Area |  |  |  |  |
| Urban | 29.4 (6.8) | 14.2 (3.0) | 13.5 (3.6) | 1.6 (0.9) |
| Rural | 29.2 (1.6) | 16.0 (1.5) | 12.2 (0.9) | 1.0 (0.4) |
|  |  |  |  |  |
|  | **Non-pregnant women, 15-49 y (n=3233)^d^** | | | |
| National | 36.3 (2.1) | 18.0 (0.8) | 16.9 (1.3) | 1.4 (0.3) |
| Region |  |  |  |  |
| West | 41.5 (2.0) | 19.6 (1.1) | 19.6 (1.3) | 2.4 (0.3) |
| Central | 36.6 (2.9) | 17.7 (1.3) | 17.7 (1.7) | 1.1 (0.2) |
| East | 30.0 (1.4) | 16.4 (1.0) | 13.2 (1.0) | 0.4 (0.1) |
| Area |  |  |  |  |
| Urban | 37.0 (2.9) | 18.3 (1.2) | 18.0 (1.9) | 0.7 (0.4) |
| Rural | 35.5 (3.8) | 17.7 (1.6) | 15.8 (2.0) | 2.1 (0.4) |
|  |  |  |  |  |
|  | **Pregnant women, 15-49 y (n=118)^e^** | | | |
| National | 28.2 (3.4) | 17.9 (3.5) | 10.3 (3.2) | 0 () |
| Region |  |  |  |  |
| West | 25.1 (4.9) | 15.9 (5.5) | 9.2 (4.3) | 0 () |
| Central | 32.2 (6.8) | 18.5 (6.2) | 13.7 (10.2) | 0 () |
| East | 28.4 (5.8) | 19.0 (5.9) | 9.4 (4.7) | 0 () |
| Area |  |  |  |  |
| Urban | 30.1 (5.4) | 20.0 (5.4) | 10.1 (4.7) | 0 () |
| Rural | 25.7 (4.5) | 15.2 (4.2) | 10.4 (5.1) | 0 () |

^a^Estimates adjusted for survey design using “svy:“ commands and for altitude using the CDC method (1989).

^b^For children, mild anemia Hb <11 g/dL; moderate Hb <10 g/dL; severe Hb <7 g/dL.

^c^For non-pregnant adolescent girls, mild anemia Hb <12 g/dL (Hb <11.5 g/dL for girls 10-11 y); moderate Hb <11 g/dL; severe Hb <8 g/dL.

^d^For non-pregnant women, mild anemia Hb <12 g/dL; moderate Hb <11 g/dL; severe Hb <8 g/dL.

^e^For pregnant women, mild anemia Hb <11 g/dL; moderate Hb <10 g/dL; severe Hb <7 g/dL.

Supplemental Table 3. Personal and household characteristics predictive of children’s risk of anemia (Hb < 11 g/dL) in survey-adjusted models (n = 1083)

| **Characteristic** | **n** | **Hb (g/dL),  Mean (SD)** | **RR (95% CI)^a^** |
| --- | --- | --- | --- |
| Region |  |  |  |
| East | 481 | 11.5 (1.4) | 1.0 |
| West | 311 | 11.7 (1.2) | 1.1 (0.9, 1.5) |
| Central | 291 | 11.6 (1.2) | 1.0 (0.7, 1.4) |
| Area |  |  |  |
| Urban | 232 | 11.6 (1.2) | 1.0 |
| Rural | 851 | 11.0 (1.3) | 0.8 (0.6, 1.0) |
| Wealth quintile |  |  |  |
| Highest | 92 | 11.7 (1.2) | 1.0 |
| High | 145 | 11.2 (1.3) | 0.9 (0.7, 1.2) |
| Medium | 196 | 11.1 (1.3) | 1.2 (0.9, 1.6) |
| Low | 312 | 11.0 (1.3) | 1.4 (1.0, 2.0) |
| Lowest | 338 | 11.0 (1.4) | 1.3 (0.9, 1.9) |
| Improved sanitation |  |  |  |
| Yes | 721 | 11.6 (1.3) | 1.0 |
| No | 362 | 10.9 (1.3) | 1.0 (0.9, 1.3) |
| Improved water |  |  |  |
| Yes | 924 | 11.6 (1.3) | 1.0 |
| No | 159 | 10.9 (1.2) | 1.1 (0.8, 1.5) |
| Age, mo |  |  |  |
| 24-59 | 709 | 11.8 (1.2) | 1.0 |
| 12-23 | 258 | 10.6 (1.3) | 2.0 (1.7, 2.5) |
| 6-11 | 116 | 10.6 (1.3) | 2.0 (1.6, 2.4) |
| Sex |  |  |  |
| Female | 575 | 11.7 (1.3) | 1.0 |
| Male | 508 | 11.0 (1.4) | 1.1 (0.9, 1.4) |
| Stunted^b^ |  |  |  |
| No | 799 | 11.6 (1.3) | 1.0 |
| Yes | 256 | 11.0 (1.3) | 1.2 (1.0, 1.4) |
| Wasted^b^ |  |  |  |
| No | 1017 | 11.6 (1.3) | 1.0 |
| Yes | 41 | 10.7 (1.3) | 1.4 (1.1, 1.8) |
| Overweight^b^ |  |  |  |
| No | 1059 | 11.6 (1.3) | 1.0 |
| Yes | 24 | 10.3 (1.4) | 1.4 (1.1, 1.8) |

^a^Multivariable models: risk ratios and confidence intervals generated with survey-adjusted Poisson regression models.

^b^Dichotomous anthropometry indicator definitions: stunted, LAZ or HAZ <-2; wasted, WLZ or WHZ <-2; underweight, WAZ<-2; overweight, WLZ or WHZ >2.

Supplemental Table 4. Personal and household characteristics predictive of adolescent girls’ risk of anemia (Hb < 12 g/dL, Hb < 11.5 g/dL for girls 10-11 y) in survey-adjusted models (n = 1216)

| **Characteristic** | **n** | **Hb (g/dL),  Mean (SD)** | **RR (95% CI)^a^** |
| --- | --- | --- | --- |
| Region |  |  |  |
| East | 521 | 13.0 (1.4) | 1.0 |
| West | 350 | 13.0 (1.6) | 1.3 (0.8, 2.2) |
| Central | 345 | 12.8 (1.5) | 1.3 (0.9, 1.8) |
| Area |  |  |  |
| Urban | 305 | 12.6 (1.5) | 1.0 |
| Rural | 911 | 13.0 (1.4) | 0.9 (0.7, 1.3) |
| Wealth quintile |  |  |  |
| Highest | 145 | 12.8 (1.4) | 1.0 |
| High | 150 | 12.6 (1.5) | 1.1 (0.8, 1.4) |
| Medium | 207 | 12.9 (1.4) | 0.7 (0.4, 1.3) |
| Low | 370 | 13.0 (1.6) | 1.2 (0.9, 1.6) |
| Lowest | 344 | 13.0 (1.3) | 1.2 (0.8, 1.8) |
| Age, y |  |  |  |
| 10-14 | 631 | 13.0 (1.3) | 1.0 |
| 15-19 | 585 | 12.8 (1.6) | 1.3 (1.0, 1.7) |
| Occupation |  |  |  |
| Student | 1045 | 13.0 (1.4) | 1.0 |
| Farmer/other job | 109 | 12.7 (1.6) | 1.4 (1.1, 1.9) |
| Other/none | 62 | 12.8 (1.6) | 1.2 (0.6, 2.2) |
| Residence |  |  |  |
| School | 225 | 13.3 (1.4) | 1.0 |
| Home | 991 | 12.8 (1.5) | 1.4 (1.0, 2.1) |

^a^Multivariable models: risk ratios and confidence intervals generated with survey-adjusted Poisson regression models.

Supplemental Table 5. Personal and household characteristics predictive of non-pregnant women’s risk of anemia (Hb < 12 g/dL) (n = 2649)

| **Characteristic** | **n** | **Hb (g/dL),  Mean (SD)** | **RR (95% CI)^a^** |
| --- | --- | --- | --- |
| Region |  |  |  |
| East | 1094 | 13.1 (1.7) | 1.0 |
| West | 798 | 12.8 (1.7) | 1.3 (1.2, 1.5) |
| Central | 757 | 12.7 (1.7) | 1.2 (1.0, 1.5) |
| Area |  |  |  |
| Urban | 648 | 12.7 (1.6) | 1.0 |
| Rural | 2001 | 12.9 (1.7) | 0.9 (0.8, 1.0) |
| Wealth quintiles |  |  |  |
| Highest | 313 | 12.8 (1.6) | 1.0 |
| High | 368 | 12.8 (1.6) | 0.8 (0.7, 1.0) |
| Medium | 506 | 12.8 (1.8) | 1.0 (0.7, 1.3) |
| Low | 791 | 13.0 (1.7) | 1.1 (0.8, 1.6) |
| Lowest | 671 | 12.9 (1.7) | 1.0 (0.7, 1.4) |
| Improved sanitation |  |  |  |
| Yes | 1910 | 12.9 (1.7) | 1.0 |
| No | 739 | 12.7 (1.8) | 1.2 (1.0, 1.4) |
| Age, y |  |  |  |
| 20-29 | 1070 | 13.0 (1.6) | 1.0 |
| 30-39 | 890 | 12.9 (1.7) | 1.3 (1.1, 1.5) |
| 40-49 | 689 | 12.7 (1.8) | 1.3 (1.0, 1.8) |
| Completed education |  |  |  |
| High school+ | 701 | 12.8 (1.6) | 1.0 |
| Primary^b^ | 729 | 13.1 (1.6) | 0.9 (0.8, 1.1) |
| None | 1214 | 12.8 (1.8) | 1.1 (0.9, 1.3) |
| Occupation |  |  |  |
| Farmer | 1300 | 13.0 (1.8) | 1.0 |
| Student | 146 | 12.7 (1.8) | 1.1 (0.7, 1.6) |
| Other employment | 392 | 12.6 (1.7) | 1.2 (1.0, 1.4) |
| Housewife/other | 811 | 12.9 (1.6) | 1.0 (0.8, 1.2) |
| Marital status |  |  |  |
| Married | 2040 | 13.0 (1.7) | 1.0 |
| Unmarried | 399 | 12.5 (1.9) | 1.3 (1.0, 1.7) |
| Separated^c^ | 210 | 12.7 (1.8) | 1.4 (1.2, 1.7) |
| Child <5 y^d^ |  |  |  |
| Yes | 949 | 13.1 (1.5) | 1.0 |
| No | 1673 | 12.7 (1.8) | 1.1 (1.0, 1.3) |

^a^Multivariable models: risk ratios and confidence intervals generated with survey-adjusted Poisson regression models.

^b^Primary category includes informal and monastic schooling.

^c^Separated category includes divorced, separated and widowed.

^d^Woman is the mother of a child age 5 y or younger.

Supplemental Table 6. Characteristics predictive of pregnant women’s odds of anemia (n =118)

| **Characteristic** | **n** | **Hb (g/dL),  Mean (SD)** | **RR (95% CI)^a^** |
| --- | --- | --- | --- |
| Region |  |  |  |
| East | 47 | 12.2 (1.4) | 1.0 |
| West | 40 | 12.2 (1.3) | 1.0 (0.5, 2.0) |
| Central | 31 | 11.7 (1.4) | 1.2 (0.5, 3.0) |
| Area |  |  |  |
| Urban | 32 | 11.9 (1.7) | 1.0 |
| Rural | 86 | 12.1 (1.3) | 0.4 (0.2, 0.9) |
| Wealth quintiles |  |  |  |
| Highest | 15 | 12.6 (1.6) | 1.0 |
| High | 23 | 12.4 (1.2) | 0.2 (0.0, 1.1) |
| Medium | 22 | 11.4 (1.5) | 4.5 (1.2, 16.5) |
| Low | 26 | 11.7 (1.1) | 6.0 (1.4, 25.5) |
| Lowest | 32 | 12.3 (1.4) | 2.7 (0.4, 17.0) |
| Age |  |  |  |
| 15-19 | 9 | 12.0 (0.6) | 1.0 |
| 20-29 | 67 | 11.8 (1.1) | 24.9 (2.0, 306.1) |
| 30-39 | 34 | 12.6 (1.8) | 18.6 (0.9, 401.3) |
| 40-49 | 8 | 11.9 (1.5) | 82.7 (4.6, 1474.3) |
| Trimester |  |  |  |
| 1st | 42 | 12.6 (1.6) | 1.0 |
| 2nd | 44 | 12.0 (1.2) | 2.6 (1.2, 5.6) |
| 3rd | 32 | 11.5 (1.2) | 2.1 (0.8, 5.3) |

^a^Multivariable models: risk ratios and confidence intervals generated with survey-adjusted Poisson regression models.

SUPPLEMENTARY REFERENCES

(1989) CDC criteria for anemia in children and childbearing-aged women. In: Morbidity and Mortality Weekly Report. Centers for Disease Control & Prevention (CDC).

Aga Khan University P., Pakistan Medical Research Council (PMRC) & Nutrition Wing C.D., Government of Pakistan, (2011) National Nutrition Survey Pakistan. Aga Khan University, Pakistan, Pakistan Medical Research Council (PMRC) and Nutrition Wing, Cabinet Division, Government of Pakistan, Karachi, Pakistan.

Department of Census and Statistics (DCS) & Ministry of Healthcare and Nutrition (MOH) (2009) Sri Lanka Demographic and Health Survey 2006-07. DCS and MOH, Colombo, Sri Lanka.

International Institute for Population Sciences - IIPS/India & Macro International (2007) National Family Health Survey (NFHS-3), 2005–06: India: Volume I. IIPS and Macro International, Mumbai, India.

Khoi HH, Khan NC, Tam NC, Mai LB, Hao LQ, Thuy PV, et al. (2001) Report on Vietnam national anemia survey, 2000. National Institute of Nutrition, Hanoi.

Ministry of Health [Lao People’s Democratic Republic] (2001) Report on national health survey: health status of the People of LAO PDR. Ministry of Health, Vientiane.

Ministry of Health and Population - MOHP/Nepal, New ERA/Nepal & ICF International (2012) Nepal demographic and health survey 2011. MOHP/Nepal, New ERA/Nepal, and ICF International, Kathmandu, Nepal.

Ministry of Health and Sports (MoHS) & ICF (2017) Myanmar demographic and health survey 2015-16. Ministry of Health and Sports and ICF, Nay Pyi Taw, Myanmar, and Rockville, Maryland USA.

Ministry of Public Health (1998) The fourth national nutrition survey of Thailand 1995. Ministry of Public Health, Department of Health, Bangkok.

Ministry of Public Health of the Islamic Republic of Afghanistan & UNICEF (2013) National Nutrition Survey Afghanistan Survey Report.

National Institute of Population Research and Training (NIPORT), Mitra and Associates & and ICF International (2013) Bangladesh demographic and health survey 2011. NIPORT, Mitra and Associates and ICF International, Dhaka, Bangladesh and Calverton, Maryland, USA.

National Institute of Statistics/Cambodia, Directorate General for Health/Cambodia & ICF International (2015) Cambodia demographic and health survey 2014. National Institute of Statistics/Cambodia, Directorate General for Health/Cambodia and ICF International, Phnom Penh, Cambodia.

Nutrition Program D.o.P.H., Ministry of Health (2015) National nutrition survey (NNS). Thimphu, Bhutan.
